# A new analytical model for bond strength between corroded steel strand and concrete

**DOI:** 10.1038/s41598-024-62763-z

**Published:** 2024-05-25

**Authors:** Hai Li, Yiming Yang, ChunHua Li, Xinzhong Wang, Huang Tang

**Affiliations:** 1 College of Civil Engineering, Hunan City University, No.518 Yinbindong Road, Yiyang, 413000 China; 2 Key Laboratory of Green Building and Intelligent Construction in Higher Educational Institutions of Hunan Province, Hunan City University, Yiyang, 413000, China; 3 Hunan Engineering Research Center of Development and Application of Ceramsite Concrete Technology, Hunan City University, Yiyang, 413000 China

**Keywords:** Bond strength, Analytical model, Strand corrosion, Concrete, Civil engineering, Computational methods

## Abstract

Degradation of bond strength due to corrosion of steel strands is of great importance for serviceability of prestressed concrete structures. An analytical model is proposed to demonstrate the effect of corrosion of steel strand on reduction of bond strength. Corrosion expansion force generated by steel strand corrosion before and after corrosion cracking is firstly estimated. Then, the reduced gripping effect of the concrete, change of friction coefficient between the corroded strand and reduction force on the bearing face are considered in calculating the pre-rib extrusion force. Finally, the enhancement of bond strength due to transverse confinement of stirrups is considered and the ultimate bond strength of corroded steel strand is calculated. Comparison of results between the prediction and experimental result shows the proposed model can be used to reasonably evaluate the bond strength. The prediction result of the bond strength model is affected by the degree of strand corrosion, but almost not by the drawing method.

## Introduction

The crack development, stiffness and bearing capacity of the pre-tensioned concrete (PC) structures are affected by the bonding behavior between steel strand and the surrounding concrete^1–3^. Because of poor construction quality of PC structure and the external erosive environment, the stands in concrete are unavoidably corroded with the increase in service time^4,5^. Corrosion products change the contact conditions and deteriorate the bond strength between steel strand and the surrounding concrete^6^, which would eventually cause degraded serviceability and insufficient load-carrying capacity in PC structures^7^. Therefore, it is necessary to understand the effect of strand corrosion on the bond mechanism, and to predict the bond strength between corroded strand and concrete.

The bond strength of steel strands embedded in concrete is similar to that of ribbed steel bars, which is mainly composed of chemical adhesive force, friction force and mechanical bite force. Because the contribution of chemical adhesive force to the bonding force is small, and it has been lost when the bonding failure occurs, the chemical adhesive force is usually ignored when analyzing the bond strength between steel strand and concrete. Many studies have shown that the bond strength between uncorroded strand and concrete is affected by many factors, including concrete compressive strength^8–10^, concrete cover^11^, type of prestressed strand^8,12^, strand position^13^, strand configuration^14,15^, strand surface condition^16,17^, and Hoyer effect^18^. In addition to the above effects, the bond strength between corroded steel strand and concrete is inevitably affected by the corrosion of steel strand. The effect of steel strand corrosion on the bond strength is similar to that of ribbed steel bar, and the corrosion products will fill the interface between strand and concrete, resulting in rust expansion effect^19,20^, changing the gripping effect of concrete on steel strand. The friction coefficient of the interface between concrete and steel strand varies with the propagation of the steel strand corrosion^21^. Corrosion also changes the pre-rib extrusion force of the steel strand, thereby affecting the friction and mechanical bite force provided by it. A few experimental investigations have been conducted to study the bonding behavior between corroded strand and concrete^19,20,22–24^.

At present, many researches have been conducted to predict the bond strength between strand and concrete. These prediction methods include three types as follow. The first type is an empirical model to estimate the bond strength between strand and concrete based on the experimental results^25,26^. The second type is to take the transfer length of prestressing strand as an indicator of bond strength. That existing models predictive bond strength by quantifying the interaction and precisely predicting the transfer length between the prestressing strands and concrete^27,28^. The third type is analytical model to predict the bond strength based on the thick-walled cylinder theory^29,30^. Compared with uncorroded strand in concrete, the research on estimating bond strength between the corroded strand and concrete is relatively less. Several empirical formulas have been established to estimate the bond strength of corroded strand in concrete members based on the experimental results^6,8,24^. Typically, these empirical formulas exhibit good agreement with some experimental results, especially the experimental results conducted by themselves. However, there is uncertainty whether they can provide acceptable predictions or not in many other experimental results. In addition, the applicability of these formulas to real structures could be limited due to potential difference between experimental tests and field conditions. Except for empirical model, most theoretical models available are concentrated on predicting bond strength of corroded deformed bars^31–33^. Beyond that, limited attention has been focused on analytical mode to predict bond strength between corroded strand and concrete. Dai et al.^34^ developed an analysis model to predict equivalent bond strength considering flexural cracks by iterative calculation of the length of slipping region. Wu^35^ analogized the effect of strand corrosion on the bonding behavior to that of corroded deformed bar, and established a theoretical analysis model based on the thick-walled cylinder theory. However, the bond for a seven-wire strand may differ from that of a deformed bar because of its twisting constitution and mechanical interlock^30^. The analytical model of bond strength between concrete and the corroded strand needs to be developed further.

Therefore, the objective of this study is to investigate the strand corrosion on friction force and mechanical bite force, which are main components of bond strength. An analytical model is proposed for predicting the bond strength between the seven-wire steel strand and concrete, taking into account the corrosion characteristics of the steel strand in concrete. The paper is organized as follows: First, calculate corrosion-induced expansive force before and after cracking through the theory of elastic thick-walled cylinder. Then, derive the pre-rib extrusion force of the corroded strand, in order to obtain the calculation model of the bond strength of the corroded strand. Subsequently, the proposed model is validated by the experimental results. Finally, several conclusions are drawn.

## Corrosion-induced expansion force generated by steel strand corrosion

**Figure 1:**
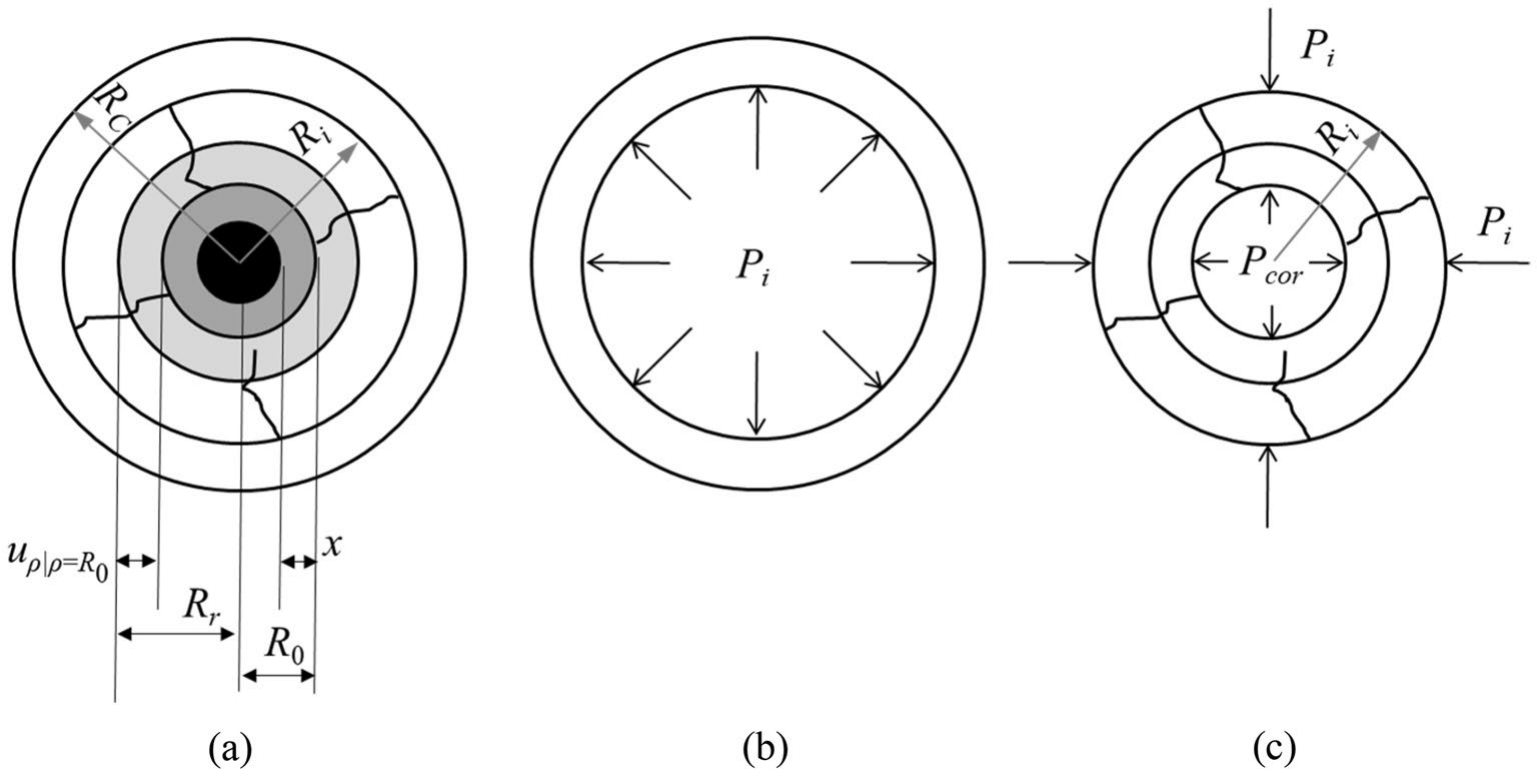
Stress distribution under corrosion-induced expansion without cracking.

The concrete around the corroded strands is regarded as a thick-walled cylinder subjected to internal pressure, and the corrosion-induced expansion is simulated by the internal pressure, as shown in Fig. 1. In the figure, *R*_0_ is the nominal radius of the uncorroded steel strand. *R*_*c*_ is the sum of the nominal radius of un-corroded steel strand and the thickness of concrete cover *c*, namely* R*_*c*_ = *R*_0_ + *c*. *R*_*i*_ is the front radius of the corrosion-induced expansion crack. At *R*_*i*_, the concrete strain reaches the concrete cracking strain *ε*_*t*_, that is, *ε*_*t*_ = *f*_*t*_/*E*_0_, where *f*_*t*_ is the tensile strength of concrete, and *E*_0_ is the elastic modulus of concrete. The effect of steel strand corrosion on *f*_*t*_ and *E*_0_ is ignored here. *R*_*r*_ is the front radius of the rust layer. *P*_*i*_ is the compressive stress between the inner crack and the outer elastomer. *P*_*cor*_ is corrosion expansion stress.* x* and *t*_*r*_ are the average corrosion depth of steel strand and the thickness of rust layer, respectively. The following assumptions are made for the bond strength analysis of corroded steel strands in concrete: (1) The corrosion expansion force generated by the steel strand corrosion is evenly distributed at the interface between the steel strand and concrete; (2) The concrete surrounding the strands is an elastomer before and after cracking.

### Effective rust thickness and critical corrosion depth

There are tiny voids in the interface between steel strands and concrete. Rust from steel strands does not produce corrosion-induced expansion at the beginning. It first fills the interfacial void, and then squeezes the surrounding concrete to produce corrosion-induced expansion force. When strand corrosion causes corrosion-induced cracks in the surrounding concrete, the rust will continue to fill the cracks. The amount of steel strand corrosion loss with an average corrosion depth *x* is Δ*V*_*s*_ = π[*R*_0_^2^−(*R*_0_−*x*)^2^], and the volume of the corresponding rust is Δ*V* = (*n*−1)Δ*V*_*s*_ = Δ*V*_1_ + Δ*V*_2_ + Δ*V*_3_. *n* is the expansion rate of the rust, which is the volume ratio of the rust products to the virgin steel. According to the different corrosion products of the steel bar, the usual value range of *n* is 2–4^36^. Δ*V*_1_ is the volume of rust filled into the voids at the interface between strand and concrete. Δ*V*_2_ is the volume of rust to produce corrosion-induced expansion force for squeezing concrete; Δ*V*_3_ is the volume of rust that fills cracks inside the concrete. According to the research of Pantazopulou^37^, Δ*V*_1_ + Δ*V*_2_ = π(*R*_r_^2^−*R*_0_^2^), Δ*V*_3_ = π*t*_*r*_(*R*_i_−*R*_r_). From this, the following equation can be established:

1 $$\Delta V = \pi \left( {R_{r}^{2} - R_{0}^{2} } \right) + \pi t_{r} \left( {R_{i} - R_{r} } \right) = \left( {n - 1} \right)\pi \left[ {R_{0}^{2} - \left( {R_{0} - x} \right)^{2} } \right]$$

The effective thickness of the rust layer *t*_*r*_ can be obtained by simplifying Eq. (1)

2 $$t_{r} = \frac{{\left( {n - 1} \right)\left( {2R_{0} x - x^{2} } \right) + x\left( {R_{i} - R_{0} + x} \right)}}{{R_{i} + R_{0} }}$$

At the radial position *ρ* = *R*_0_, the radial corrosion-induced expansion displacement could be determined that

3 $$u_{\rho } \left| {_{{\rho = R_{0} }} = R_{r} } \right. - R_{0} = t_{r} - x = \frac{{\left( {n - 1} \right)\left( {2R_{0} x - x^{2} } \right)}}{{R_{i} + R_{0} }}$$

Considering the uncracked outer ring elastic part in Fig. 1b, the method of solving the displacement of the thick-walled cylinder in elasticity^38^ can be employed to calculate the axisymmetric stress and displacement of the annular force.

4 $$\sigma_{\rho } = \frac{A}{{\rho^{2} }} + 2C,\;\;\sigma_{\varphi } = - \frac{A}{{\rho^{2} }} + 2C,\;\;\tau_{\rho \varphi } = \tau_{\varphi \rho } = 0$$

5 $$u_{\rho } = \frac{1}{{E_{c} }}\left[ { - \left( {1 + \nu } \right)\frac{A}{\rho } + 2\left( {1 - \nu } \right)C\rho } \right],\;\;u_{\varphi } = 0$$

where *σ*_*ρ*_ and *σ*_*φ*_ are the radial and circumferential stresses respectively; *τ*_*ρφ*_ and *τ*_*φρ*_ are shear stresses; *E*_*c*_ and *ν* are the elastic modulus and Poisson's ratio of the material respectively; *A* and *C* are undetermined constants. The radial stress at the radius of the crack front is the concrete tensile strength *f*_*t*_*,* and the radial stress at the outer side of the protective layer is zero. The radial displacement at the radial distance* ρ* (*R*_*i*_ ≤ *ρ* ≤ *R*_*c*_) can be obtained as follow.

6 $$\left( {\sigma_{\rho } } \right)_{{\rho { = }R_{c} }} = \frac{A}{{R_{c}^{2} }} + 2C = 0,\;\;\left( {\sigma_{\varphi } } \right)_{{\rho { = }R_{i} }} = - \frac{A}{{R_{i}^{2} }} + 2C{ = }f_{t}$$

Through Eq. (6), the constants *A* and *C* can be determined.

7 $$A = \frac{{ - f_{t} R_{i}^{2} R_{c}^{2} }}{{R_{i}^{2} + R_{c}^{2} }},\;\;\;\;\;\;\;C = \frac{1}{2}\frac{{f_{t} R_{i}^{2} }}{{R_{i}^{2} + R_{c}^{2} }}$$

Substitute Eq. (7) into Eq. (5), the radial displacement at *ρ* (*R*_*i*_ ≤ *ρ* ≤ *R*_*c*_) can be calculated by

8 $$u_{\rho } = \frac{{f_{t} \rho }}{{E_{c} }}\left\{ {\frac{{\left( {{{R_{c} } \mathord{\left/ {\vphantom {{R_{c} } \rho }} \right. \kern-0pt} \rho }} \right)^{2} + 1}}{{\left( {{{R_{c} } \mathord{\left/ {\vphantom {{R_{c} } {R_{i} }}} \right. \kern-0pt} {R_{i} }}} \right)^{2} + 1}} + \frac{{\nu \left[ {\left( {{{R_{c} } \mathord{\left/ {\vphantom {{R_{c} } \rho }} \right. \kern-0pt} \rho }} \right)^{2} - 1} \right]}}{{\left( {{{R_{c} } \mathord{\left/ {\vphantom {{R_{c} } {R_{i} }}} \right. \kern-0pt} {R_{i} }}} \right)^{2} + 1}}} \right\}$$

Because the circumferential stress is obviously smaller than the radial stress, the radial displacement caused by the circumferential tensile stress after concrete cracking can be ignored. Therefore, the Poisson effect is not considered when calculating the radial displacement, that is, *ν* = 0 in Eq. (8). The radial displacement at *ρ* in Eq. (8) could be determined by

9 $$u_{\rho } = \frac{{f_{t} \rho }}{{E_{c} }}\frac{{\left( {{{R_{c} } \mathord{\left/ {\vphantom {{R_{c} } \rho }} \right. \kern-0pt} \rho }} \right)^{2} + 1}}{{\left( {{{R_{c} } \mathord{\left/ {\vphantom {{R_{c} } {R_{i} }}} \right. \kern-0pt} {R_{i} }}} \right)^{2} + 1}}$$

Corresponding to the cracked inner ring of Fig. 1c, the circumferential stress gradually decreases from *f*_*t*_ in the radial direction and reaches the minimum value at the position* ρ* = *R*_0_. In order to calculate the circumferential strain of the cracked inner ring, both the uncracked outer ring and the cracked inner ring obey the assumption of elastomer in the range of *R*_0_ ≤ *R*_*i*_ ≤ *R*_*c*_. According to the Eq. (9), the radial displacement at *ρ* = *R*_0_ could be calculated by

10 $$u_{{\rho = R_{0} }} = \frac{{f_{t} R_{0} }}{{E_{c} }}\frac{{\left( {{{R_{c} } \mathord{\left/ {\vphantom {{R_{c} } {R_{0} }}} \right. \kern-0pt} {R_{0} }}} \right)^{2} + 1}}{{\left( {{{R_{c} } \mathord{\left/ {\vphantom {{R_{c} } {R_{i} }}} \right. \kern-0pt} {R_{i} }}} \right)^{2} + 1}}$$

From Eqs. (4) and (10), it can be obtained that

11 $$\frac{{\left( {n - 1} \right)\left( {2R_{0} x - x^{2} } \right)}}{{R_{i} + R_{0} }} = \frac{{f_{t} R_{0} }}{{E_{c} }}\frac{{\left( {{{R_{c} } \mathord{\left/ {\vphantom {{R_{c} } {R_{0} }}} \right. \kern-0pt} {R_{0} }}} \right)^{2} + 1}}{{\left( {{{R_{c} } \mathord{\left/ {\vphantom {{R_{c} } {R_{i} }}} \right. \kern-0pt} {R_{i} }}} \right)^{2} + 1}}$$

Because *R*_0_ ≤ *R*_*i*_ ≤ *R*_*c*_ , the average corrosion depth of different corrosion front radius can be obtained from Eq. (11). When *R*_*i*_ = *R*_*c*_, the critical corrosion depth *x*_*cr*_ for the protective layer cracking can be obtained.

### Corrosion-induced expansion force without cracking

**Figure 2:**
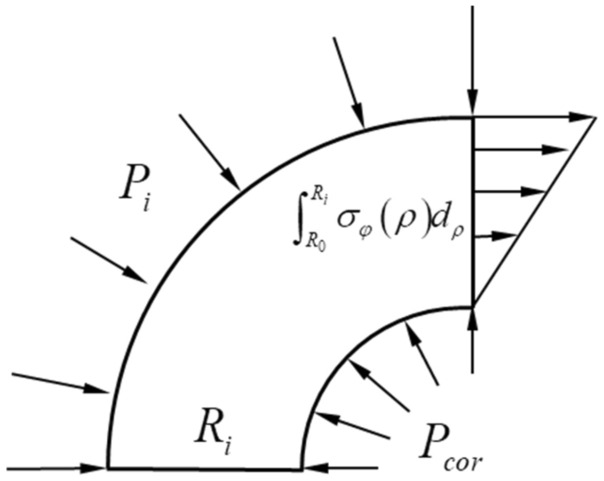
Schematic diagram of the force on the crack front.

Figure 2 is a schematic diagram of the force on the front edge of the crack when the protective layer is not cracked. From the balance conditions of the force, it can be expressed as

12 $$P_{i} R_{i} + \int {_{{R_{0} }}^{{R_{i} }} } \sigma_{\varphi } \left( \rho \right)d\rho = P_{cor} R_{0}$$

where *σ*_*φ*_(*ρ* = *R*_*i*_) = *f*_*t*_ is given in the elastic part of the outer loop, *P*_*i*_ is obtained from Eqs. (6) and (7). (where *P*_*i*_ can be obtained by setting *σ*_*φ*_(*ρ* = *R*_*i*_) = *f*_*t*_ in the elastic part of the outer loop (Fig. 1c)).

13 $$P_{i} = \left( {\sigma_{\varphi } } \right)_{{\rho = {\text{R}}_{i} }} = f_{t} \frac{{R_{c}^{2} - R_{i}^{2} }}{{R_{c}^{2} + R_{i}^{2} }}$$

In order to determine the circumferential stress of the cracked inner ring, the relationship between the circumferential stress and the related radial displacement is considered first. The total circumferential deformation at the radial radius *ρ* of the inner ring is expressed as

14 $$2\pi \rho \varepsilon_{\varphi } \left( \rho \right) = 2\pi u_{\rho }$$

where *u*_*ρ*_ can be obtained from Eq. (9). If the average corrosion rate or average corrosion depth *x* is known, the corrosion front radius *R*_*i*_ can be found from Eq. (11), *ε*_*φ*_(*ρ*) is concluded by

15 $$\varepsilon_{\varphi } \left( \rho \right){ = }\frac{{u_{\rho } }}{\rho }{ = }\frac{{f_{t} }}{{E_{c} }}\frac{{({{R_{c} } \mathord{\left/ {\vphantom {{R_{c} } {\rho )^{2} + 1}}} \right. \kern-0pt} {\rho )^{2} + 1}}}}{{({{R_{c} } \mathord{\left/ {\vphantom {{R_{c} } {R_{i} )^{2} + 1}}} \right. \kern-0pt} {R_{i} )^{2} + 1}}}}$$

**Figure 3:**
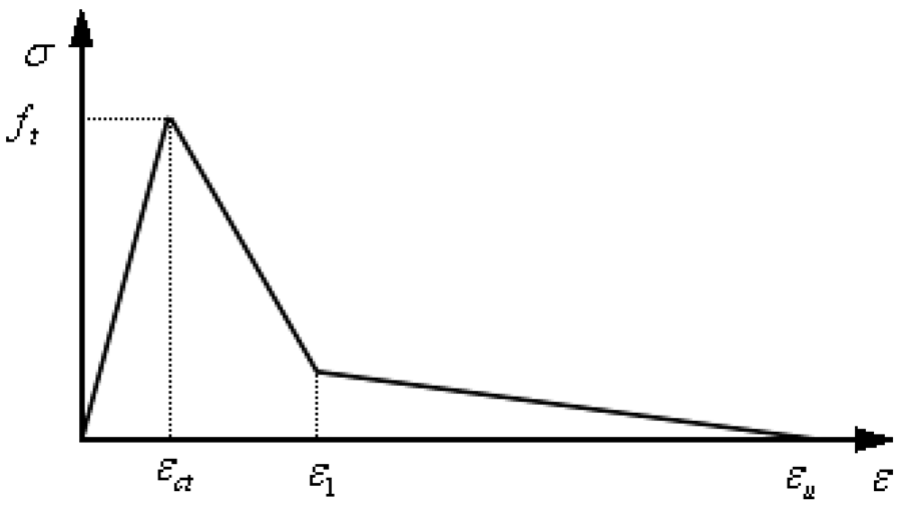
Tensile stress–strain curve of concrete.

There is *R*_0_ ≤ *ρ* ≤ *R*_*i*_ in cracked inner ring, then *ε*_*φ*_(*ρ*) ≥ *f*_*t*_ /*E*_0_ = *ε*_*ct*_. As the circumferential strain of concrete is greater than the concrete cracking strain, it can be assumed that a diffuse crack is generated in the radial direction. Considering the softening properties of concrete, the average circumferential stress and average circumferential strain of concrete under tensile action can be described by Fig. 3, and the specific relationship is expressed as^37^

16 $$\sigma_{\varphi } \left( \rho \right) = E_{c} \varepsilon_{\varphi } \left( \rho \right),\;\;\varepsilon_{\varphi } \left( \rho \right) \le \varepsilon_{ct}$$

17 $$\sigma_{\varphi } \left( \rho \right) = f_{t} \left[ {1 - 0.85\frac{{\varepsilon_{\varphi } \left( \rho \right) - \varepsilon_{ct} }}{{\varepsilon_{1} - \varepsilon_{ct} }}} \right],\;\;\varepsilon_{ct} < \varepsilon_{\varphi } \left( \rho \right) \le \varepsilon_{1}$$

18 $$\sigma_{\varphi } \left( \rho \right) = 0.15f_{t} \frac{{\varepsilon_{u} - \varepsilon_{\varphi } \left( \rho \right)}}{{\varepsilon_{u} - \varepsilon_{1} }},\;\;\varepsilon_{1} < \varepsilon_{\varphi } \left( \rho \right) \le \varepsilon_{u}$$

Before concrete cracking, the stress *σ*_*φ*_(*ρ*) and strain *ε*_*φ*_(*ρ*) shows a linear elastic relationship, and the stress–strain slope is *Ec*. After concrete cracking, the concrete begins to soften, and *ε*_*φ*_(*ρ*) ≥ *ε*_*ct*_ . The stress–strain relationship curve finally terminates at *ε*_*φ*_(*ρ*) ≥ *ε*_*u*_, where *ε*_*u*_ is the ultimate strain of the concrete. In Fig. 3, *ε*_1_ and *ε*_*u*_ take 0.0003 and 0.002 respectively. To find $\int {_{{R_{0} }}^{{R_{i} }} } \sigma_{\varphi } \left( \rho \right)d\rho$, *R*_1_ and *R*_u_ are defined firstly as^39^

19 $$R_{1} = \frac{{R_{c} }}{{\sqrt {{{\varepsilon_{1} E_{c} } \mathord{\left/ {\vphantom {{\varepsilon_{1} E_{c} } {f_{t} \left[ {{{\left( {R_{c} } \right.} \mathord{\left/ {\vphantom {{\left( {R_{c} } \right.} {\left. {R_{i} } \right)^{2} + 1}}} \right. \kern-0pt} {\left. {R_{i} } \right)^{2} + 1}}} \right] - 1}}} \right. \kern-0pt} {f_{t} \left[ {{{\left( {R_{c} } \right.} \mathord{\left/ {\vphantom {{\left( {R_{c} } \right.} {\left. {R_{i} } \right)^{2} + 1}}} \right. \kern-0pt} {\left. {R_{i} } \right)^{2} + 1}}} \right] - 1}}} }}$$

20 $$R_{u} = \frac{{R_{c} }}{{\sqrt {{{\varepsilon_{u} E_{c} } \mathord{\left/ {\vphantom {{\varepsilon_{u} E_{c} } {f_{t} \left[ {{{\left( {R_{c} } \right.} \mathord{\left/ {\vphantom {{\left( {R_{c} } \right.} {\left. {R_{i} } \right)^{2} + 1}}} \right. \kern-0pt} {\left. {R_{i} } \right)^{2} + 1}}} \right] - 1}}} \right. \kern-0pt} {f_{t} \left[ {{{\left( {R_{c} } \right.} \mathord{\left/ {\vphantom {{\left( {R_{c} } \right.} {\left. {R_{i} } \right)^{2} + 1}}} \right. \kern-0pt} {\left. {R_{i} } \right)^{2} + 1}}} \right] - 1}}} }}$$

where *R*_1_ and *R*_*u*_ are the radial distances when the ring strain equals *ε*_1_ and *ε*_u_. According to the size relationship between *R*_0_, *R*_1_ and *R*_*u*_, $\int {_{{R_{0} }}^{{R_{i} }} } \sigma_{\varphi } \left( \rho \right)d\rho$ can be obtained. When the protective layer is not cracked (*x* ≤ *x*_*cr*_), corrosion-induced expansion force *P*_*cor*_ at different corrosion depths can be obtained from Eq. (12).

### Corrosion-induced expansion force with cracking (*x* > *x*_*cr*_)

When the average corrosion depth *x* is greater than the critical corrosion depth *x*_*cr*_, the concrete protective layer is completely cracked. After the corrosion-induced expansion cracking of the protective layer can be obtained by substituting *R*_*i*_ = *R*_*c*_ into the Eq. (3), the radial corrosion expansion-induced displacement at the radial position *x* can be calculated by

21 $$u_{{\rho = R_{0} }} = \frac{{\left( {n - 1} \right)\left( {2R_{0} x - x^{2} } \right)}}{{R_{c} + R_{0} }}$$

*P*_*i*_ = 0 and *R*_*i*_ = *R*_*c*_ in Eq. (12) results in

22 $$\int {_{{R_{0} }}^{{R_{c} }} } \sigma_{\varphi } \left( \rho \right)d\rho = p_{cor} R_{0}$$

To obtain *σ*_*φ*_(*ρ*) in Eq. (22), the radial displacement within *R*_0_ ≤ *ρ* ≤ *R*_*c*_ of the entire cracking range of the protective layer needs to be considered. *f*_*t*_ /*E*_*c*_ in Eq. (9) is the circumferential strain when *ρ* = *R*_*i*_. Assuming that the circumferential strain of *ρ* = *R*_*c*_ becomes *ε*_*φc*_, *ε*_*φc*_ is substituted for *f*_*t*_ /E_c_ in Eq. (9), and *R*_*i*_ = *R*_*c*_ is given to obtain the radial displacement in the entire cracking range *R*_0_ ≤ *ρ* ≤ *R*_*c*_.

23 $$u_{\rho } = \varepsilon_{\varphi c} \rho \frac{{\left( {{{R_{c} } \mathord{\left/ {\vphantom {{R_{c} } \rho }} \right. \kern-0pt} \rho }} \right)^{2} + 1}}{2}$$

From the deformation coordination conditions at the radial distance *ρ* in Eq. (15), *σ*_*φ*_(*ρ*) can be obtained as

24 $$\varepsilon_{\varphi } \left( \rho \right){ = }\frac{{u_{\rho } }}{\rho }{ = }\varepsilon_{\varphi c} \frac{{({{R_{{\text{c}}} } \mathord{\left/ {\vphantom {{R_{{\text{c}}} } {\rho )^{2} + 1}}} \right. \kern-0pt} {\rho )^{2} + 1}}}}{2}$$

where *ε*_*ct*_ < *ε*_*φ*_(*ρ*) ≤ *ε*_*u*_.

When the corrosion depth *x* is known, *ε*_*φc*_ can be obtained by the coordinated deformation of Eqs. (21) and (24) at *ρ* = *R*_0_.

25 $$\varepsilon_{\varphi c} = \frac{{\left( {n - 1} \right)\left( {2R_{0} x - x^{2} } \right)}}{{R_{c} + R_{0} }}\frac{2}{{R_{0} }}\frac{1}{{({{R_{{\text{c}}} } \mathord{\left/ {\vphantom {{R_{{\text{c}}} } {R_{0} )^{2} + 1}}} \right. \kern-0pt} {R_{0} )^{2} + 1}}}}$$

To find $\int {_{{R_{0} }}^{{R_{c} }} } \sigma_{\varphi } \left( \rho \right)d\rho$, *R*_1_ and *R*_*u*_ are also defined as follows.

26 $$R_{1} = \frac{{R_{{\text{c}}} }}{{\sqrt {{{2\varepsilon_{1} } \mathord{\left/ {\vphantom {{2\varepsilon_{1} } {\varepsilon_{\varphi c} - 1}}} \right. \kern-0pt} {\varepsilon_{\varphi c} - 1}}} }}$$

27 $$R_{u} = \frac{{R_{{\text{c}}} }}{{\sqrt {{{2\varepsilon_{u} } \mathord{\left/ {\vphantom {{2\varepsilon_{u} } {\varepsilon_{\varphi c} - 1}}} \right. \kern-0pt} {\varepsilon_{\varphi c} - 1}}} }}$$

where *R*_1_ and *R*_*u*_ are the radial distances when the circumferential strain is equal to* ε*_1_ and *ε*_*u*_. According to the magnitude relationship between *R*_0_, *R*_1_ and *R*_*u*_, $\int {_{{R_{0} }}^{Rc} } \sigma_{\varphi } \left( \rho \right)d\rho$ can be obtained. As the protective layer is cracked (*x* > *x*_*cr*_), the corrosion-induced expansion force *P*_*cor*_ at different corrosion depths can be obtained from Eq. (22).

## Calculation of pre-rib extrusion force under strand corrosion

**Figure 4:**
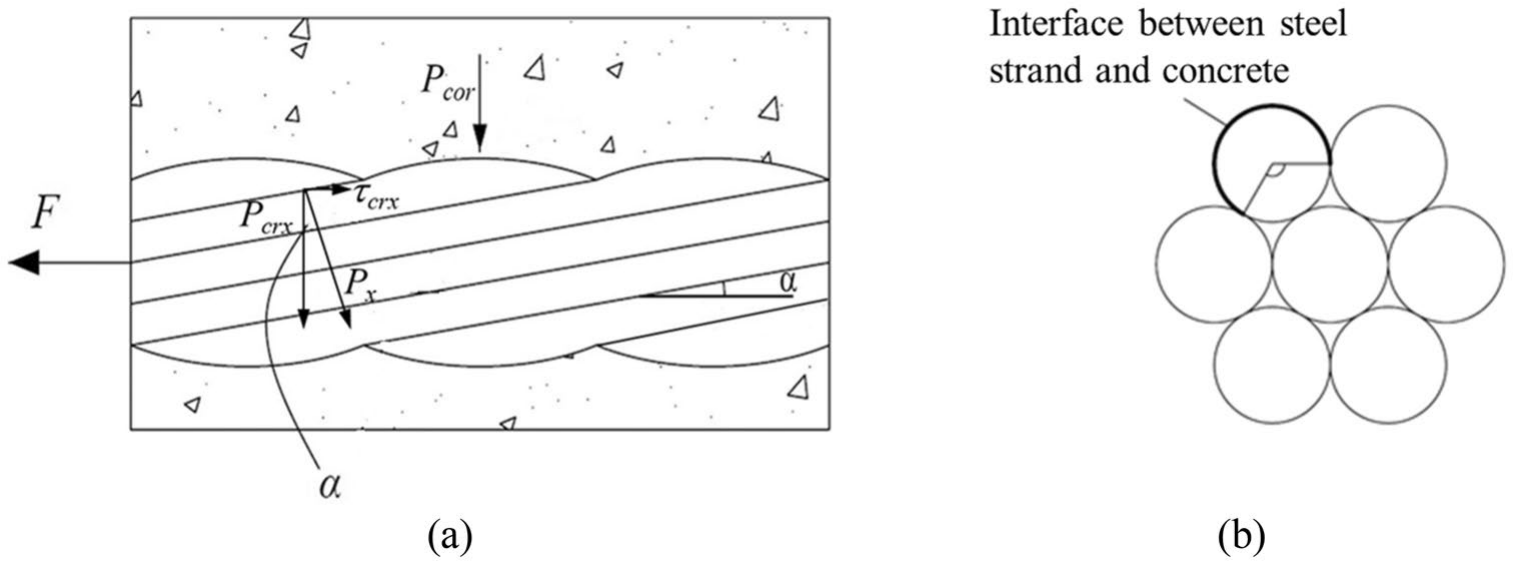
Analysis diagram of the bonding force between steel strands and concrete.

In prestressed concrete with steel strand, the bonding force provided by the mechanical bite force mainly comes from the extrusion force generated by the twisted strands when they are stressed, that is, the pre-rib extrusion force *P*_*x*_. The horizontal component of the pre-rib extrusion force $\tau$_*crx*_ is the mechanical bite force between the steel strand and the concrete, and the vertical component force *P*_*crx*_ constitutes the partial gripping friction between the steel strand and the concrete. In order to obtain the adhesion between the steel strand and the concrete, the pre-rib extrusion force *P*_*x*_ should be obtained first. Figure 4 is a diagram of the extrusion effect between steel strand and concrete.

**Figure 5:**
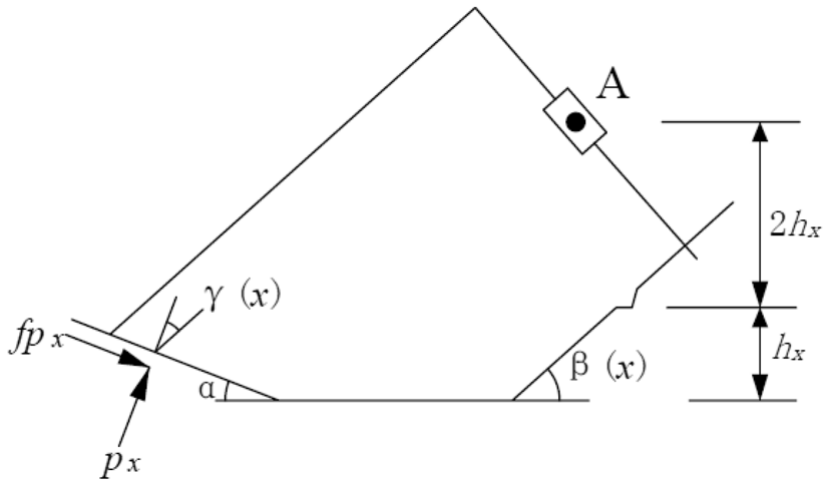
Schematic diagram of the development of oblique crack at the rib top.

When the steel strand is slid under force, the rib back pulls off to form an oblique crack at the rib top, as shown in Fig. 5. Oblique cracks stop developing at about twice the rib height^40^, at which point invisible micro cracks and oblique cracks cut the gripped concrete, forming a series of compressed conical cylinders. The stress–strain state at the top of the cylinder was analyzed, and the pre-rib extrusion force under the critical state of the limit bonding force was determined by using the multiaxial strength criterion.

According to the formula proposed by Xu^40^, the inclination angle of the pre-rib oblique crack of uncorroded reinforced concrete *β* is determined by

28 $$\beta { = }\frac{1}{2}{\text{arctg}}\left[ { - \frac{{2\left( {\sin \varphi + \mu \cos \varphi } \right)}}{\cos \varphi - \mu \sin \varphi }} \right] + 90^{ \circ }$$

where *φ* is the inclination angle of the extrusion surface, and the seven-wire strand takes *φ* = 60°; The *μ* is the friction coefficient between corroded steel strand and concrete, and the *μ* = 0.3 when it is not corroded^40^. Considering the influence of corrosion on the surface of the strand, the friction coefficient *μ*(*x*) is determined by^21^

29 $$\mu \left( x \right){ = 0}{\text{.37}} - {0}{\text{.26}}\left( {x - x_{cr} } \right)$$

Substituting Eq. (29) into Eq. (28) yields the inclination angle of the pre-rib oblique crack under different corrosion depths* β* (*x*). The angle between the pre-rib extrusion force *P*_*x*_ and the oblique crack *γ*(*x*) can be obtained by the geometric relationship: *γ*(*x*) = 90°−*α*−*β*(*x*), where* α* is the inclination angle of the cone.

It is assumed that oblique cracks with an inclination of *β* develop to stagnation at twice the rib height^40^. The average radial stress *σ*_*c*_(*x*) and shear stress *τ*(*x*) of the root of the concrete occlusal tooth (point A) after steel corrosion can be obtained from the geometric relationship^40^.

30 $$\sigma_{c} \left( x \right) = \left( {\cos \gamma \left( x \right) + f \cdot \sin \gamma \left( x \right)} \right)P_{x} \cdot \frac{{\pi (d_{0} + h_{x} )h_{x} /\sin \alpha }}{{\pi (d_{0} + 6h_{x} )l_{r} \sin \beta \left( x \right)}}$$

31 $$\tau \left( x \right) = \left( { - \sin \gamma \left( x \right) + f \cdot \cos \gamma \left( x \right)} \right)P_{x} \cdot \frac{{\pi (d{}_{0} + h_{x} )h_{x} /\sin \alpha }}{{\pi (d{}_{0} + 6h_{x} )l_{r} \sin \beta \left( x \right)}}$$

where *d*_0_ is the core radius of the steel strand, take *d*_0_ = 0.96*d*; *l*_*r*_ is rib spacing for ribbed rebar, and *l*_*r*_ can be taken as the outer arc spacing of the steel strands for steel strands; *f* is the friction coefficient between concrete crushing accumulation and concrete, regardless of the influence of steel corrosion, and the suggested value of *f* is *f* = 0.6^40^; *h*_*x*_ is the average rib height of the rebar, and takes 0.02*d* for the uncorroded ribbed elliptical rebar, where* d* is the diameter of the uncorroded rebar. The shape of uncorroded seven-wire strand is similar to that of ribbed elliptical steel bar^41^, so the value of *h*_*x*_ is the same for steel strand as that of ribbed elliptical steel bar.

When the corrosion depth of the steel strand is *x*, the equivalent diameter of the steel strand is *d*_*x*_ = *d*−2*x*, then *h*_*x*_ = 0.02*d*_*x*_. Average radial pressure *P*_*crx*_ at the interface is calculated by

32 $$P_{crx} = (\cos \alpha - f\sin \alpha )P_{x} \frac{{\pi \left( {d_{0} + h_{x} } \right)h_{x} /\sin \alpha }}{{\pi d_{x} l_{r} }}$$

The circumferential average tensile stress induced by the radial pressure *P*_*crx*_ is expressed as

33 $$\sigma_{\varphi } \left( x \right) = \frac{10}{9}\left( {1 - \frac{{{{d_{0} } \mathord{\left/ {\vphantom {{d_{0} } 2}} \right. \kern-0pt} 2} + 3h_{x} }}{{5d_{x} }}} \right)\frac{1}{{2\frac{{C_{e} }}{{d_{x} }}\left( {1 - \frac{{C_{e} }}{{9d_{x} }}} \right)}}P_{crx}$$

where *C*_*e*_ is the effective protective layer thickness of concrete; It mainly considers the weakening of the gripping effect on the concrete protective layer due to strand corrosion, and is divided into two situations before and after corrosion-induced cracking. *C*_*e*_ before the corrosion-induced cracking can be calculated by

34 $$C_{e} = \left\{ \begin{gathered} R_{c} - R_{0} {\kern 1pt} {\kern 1pt} = c,{\kern 1pt} {\kern 1pt} {\kern 1pt} {\kern 1pt} {\kern 1pt} {\kern 1pt} {\kern 1pt} {\kern 1pt} {\kern 1pt} {\kern 1pt} {\kern 1pt} {\kern 1pt} {\kern 1pt} {\kern 1pt} \;\;\;\;R_{u} \le R_{0} \hfill \\ R_{c} - R_{u} ,\;\;\;\;\;\;\;\;\;\;\;\;\;R_{0} \le R_{u} \hfill \\ \end{gathered} \right.$$

where *R*_*c*_ and *R*_*u*_ are correspond to the calculation formula before corrosion expansion cracking; They can be calculated from Eqs. (19) and (20), respectively.

After corrosion-induced cracking, it is assumed that the effective protective layer thickness is related to the average corrosion rate *η*_*p*_ of the steel strand and the minimum effective protective layer thickness *C*_*e*min_. where *C*_*e*min_ = *R*_*c*_-*R*_*u*max_, *R*_*u*max_ is obtained by *R*_*i*_ = *R*_*c*_ in Eq. (20), and the effective thickness of the protective layer after corrosion-induced cracking is deduced from the test data fitting, which can be expressed as

35 $$C_{e} = \left( {1.36 - 6.67\eta_{p} } \right)C_{e\min }$$

From the Eqs. (30)–(33), the stress at the root (failure surface) of the occlusal tooth A can be obtained, and the three principal stresses *σ*_1x_, *σ*_2x_ and *σ*_3x_ are determined by

36 $$\sigma_{1x} = - \frac{{\sigma_{c} \left( x \right)}}{2} + \sqrt {\left( {\frac{{\sigma_{c} \left( x \right)}}{2}} \right)^{2} + \tau \left( x \right)^{2} }$$

37 $$\sigma_{2x} = \sigma_{\varphi } \left( x \right)$$

38 $$\sigma_{3x} = - \frac{{\sigma_{c} \left( x \right)}}{2} - \sqrt {\left( {\frac{{\sigma_{c} \left( x \right)}}{2}} \right)^{2} + \tau \left( x \right)^{2} }$$

The failure conditions of concrete under multiaxial stress state are calculated according to Hsieh-Ting-Chen criterion^42^.

39 $$k_{1} \frac{{J_{2} }}{{f_{{\text{c}}}^{{2}} }} + k_{2} \frac{{\sqrt {J_{2} } }}{{f_{{\text{c}}} }} + k_{3} \frac{{\sigma_{1} }}{{f_{{\text{c}}} }} + k_{4} \frac{{I_{1} }}{{f_{{\text{c}}} }} - 1 = 0$$

where *σ*_1_ is the first principal stress; *I*_1_ and *J*_2_ are the first invariant and the second invariant of the stress tensor, respectively. *f*_*c*_ is the axial compressive strength of concrete; The parameters *k*_1_ = 2.0101, *k*_2_ = 0.9714, *k*_3_ = 9.1412, *k*_4_ = 0.2312 are determined by multiaxial strength test.

Substituting Eqs. (30)–(38) into Eq. (39), the pre-rib extrusion force *P*_*x*_ can be obtained from the failure conditions.

## Bond strength between corroded steel strand and concrete

### Without transverse constraint of stirrups

The adhesion force *τ*_*crx*_ provided by the extrusion force can be obtained by the pre-rib extrusion force *P*_*x*_ and the projecting horizontally from the corresponding friction resistance *μ*(x) *P*_*x*_.

40 $$\tau_{crx} = (\sin \alpha + f \cdot \cos \alpha )P_{x} \cdot \frac{{\pi (d{}_{0} + h_{x} )h_{x} /\sin \alpha }}{{\pi d{}_{0}l_{r} }}$$

It is assumed that the direction of the corrosion expansion force *P*_*cor*_ is consistent with the direction of *P*_*cr*_(*x*), and the bond strength of the splitting failure between corroded strand and concrete can be derived from

41 $$\tau_{cr} \left( x \right) = \tau_{crx} + \tan \alpha P_{cor}$$

### Transverse constraint of stirrups

Stirrups can effectively improve the bond strength of corroded steel stranded concrete^43^, because the stirrups can increase the gripping effect of concrete. In order to consider the increase effect of stirrups, it is multiplied to enhancement coefficient *K*_*sv*_ on the bond strength without stirrups transverse constraint.

42 $$K_{sv} = 1 + 8.5\rho_{sv}$$

where *ρ*_*sv*_ is the stirrup rate of the splitting surface, and the specific calculation could be calculated by

43 $$\rho_{sv} = \frac{{A_{sv} }}{{c_{e} s_{sv} }} = \frac{{\pi d_{sv}^{2} }}{{4c_{e} s_{sv} }}$$

where *A*_*sv*_ is the stirrup area; *c*_*e*_ is the thickness of effective protective layer, which is calculated according to Eqs. (34) and (35); *s*_*sv*_ is stirrup spacing; *d*_*sv*_ is the stirrup diameter. The ultimate bond strength *τ*_*u*_(*x*) of corroded steel strand with transverse confinement of stirrups can be calculated by

44 $$\tau_{u} \left( x \right) = K_{sv} \tau_{cr} \left( x \right) = K_{sv} \left( {\tau_{crx} + tg\alpha P_{cor} } \right)$$

### Calculation flowchart of bond strength between corroded strand and concrete

**Figure 6:**
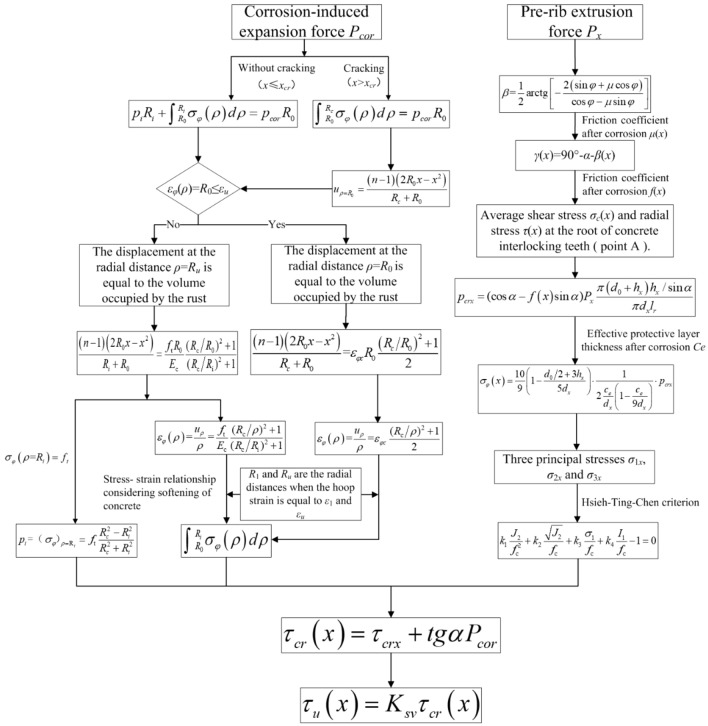
Flowchart of calculation of bond strength between corroded strand and concrete.

The calculation process of the total bond strength between corroded strand and concrete is shown in Fig. 6.

Step1: According to the equal volume of the rust, the effective rust layer thickness *t*_*r*_ is obtained. The critical corrosion depth *x*_*cr*_ for the protective layer cracking can be obtained from the consistent radial displacement distribution between the un-cracked outer ring and the cracked inner ring.

Step2: Calculate corrosion-induced expansion *P*_*cor*_ force before and after the corrosion expansion cracking of the protective layer.

Step3: The relationship between pre-rib extrusion force *P*_*x*_ and frictional resistance of the strand rib and the stress state on the failure surface is found out by the geometric conditions. The failure condition of concrete is determined multiaxial stress state, and then pre-rib extrusion force *P*_*x*_ under the critical state of the ultimate bonding force is obtained.

Step4: Calculate the bond strength between corroded strand and concrete without stirrups according to the corrosion-induced expansion *P*_*cor*_ force and pre-rib extrusion force *P*_*x*_.

Step5: Obtain the bond strength between corroded strand and concrete considering the transverse constraint of stirrups.

## Model validation

**Figure 7:**
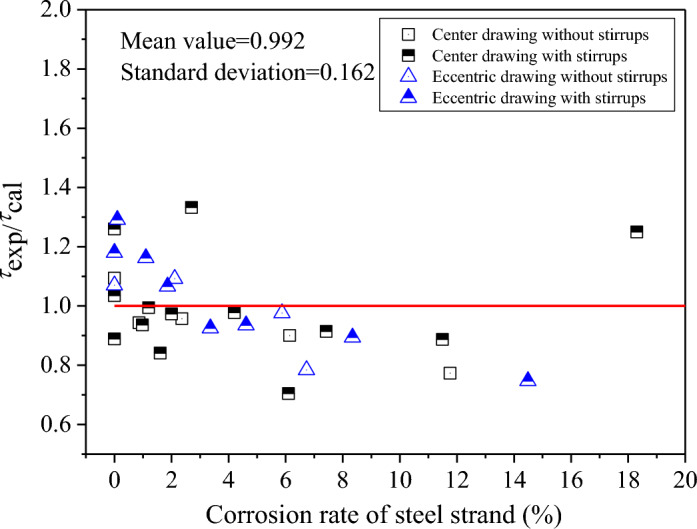
The relationship between the corrosion rate of steel strand and the ratio of the test value of the ultimate bond strength to the calculated value.

The experimental results, conducted by the authors^6^ and Wu^35^, are selected to verify the proposed model. All these test results were based on the pull-out test with a short embedment length. Table 1 gives the specimen details in the experiments, including the corrosion rate of steel strand (*η*_*p*_), the nominal steel strands diameter (*R*_0_), effective bond length (*l*_*eb*_), minimum concrete cover depth (*c*), compressive strength of concrete (*f*_*cu*_), stirrup diameter (*d*_*sv*_) and spacing (*S*_*sv*_). The basic parameters used for the calculation of the analytical model are as follows: *R*_0_ = 7.6 mm (equivalent radius of steel strand), *R*_*c*_ = *c* (radial radius of protective layer), *α* = 12.5°, *β* = 60° (angle of inter-ribbed extrusion surface), *l*_*r*_ = 7.6 mm (inter-ribbed distance), *f* = 0.6 (friction coefficient between broken accumulation and concrete). The conversion relations between *f*_*cu*_ (cube compressive strength of concrete), *f*_*c*_ (axial compressive strength of concrete) and *f*_*t*_ (axis tensile strength of concrete) are given in GB50010-2010^44^, as shown in Table 2. Substituting the above parameters, the comparison results between the ultimate average bond strength according to the calculation process of Fig. 6 and the experimental measured values under different corrosion rates of steel strands are obtained, as shown in Table 3 and Fig. 7. It can be seen from Table 3 and Fig. 7 that the average ratio of the experimental value of the ultimate bond strength to the calculated value of the analytical model is 0.992, and the coefficient of variation is 0.162. The experimental value is slightly larger than the calculated value, and the analytical model can reasonably predict the ultimate bond strength of corroded steel strand in concrete. As the strand corrosion exhibited beneficial effects on the bond strength when the corrosion loss was less than 6.24%^20^, the following is a discussion of the calculation results of the model for two types of bonded specimens with steel strand corrosion rates greater than or less than 6.24%. As shown in Fig. 7, it can be seen that when the corrosion rate of steel strands (*ƞ*_*p*_) is less than 6.24%, the predicted value of the model is small, and the average ratio of the experimental value to the calculated value is 1.023. However, when *ƞ*_*p*_ ≥ 6.24%, the average value of the ratio is 0.891, and the test value is less than the calculated value, indicating that the analytical model overestimates the ultimate bond strength in the cracking specimen. The reason may be that although the stress–strain relationship of the cracked concrete is considered in the calculation process, some calculations after cracking are proposed according to the linear elastic conditions, which are different from the actual stress characteristics. From Fig. 7, it can also be obtained that the ratio of the measured value to the calculated value is 0.981 and 1.009 under center tension and eccentric tension, respectively. The difference between the two is small, that is, the above analytical model is not sensitive to the influence of the drawing method.

**Table 1:** Specimen details in the experiments.

Literatures	*η*_*p*_ (%)	*R*_0_ (mm)	*l*_*eb*_ (mm)	*c* (mm)	*f*_*cu*_ (Mpa)	*d*_*sv*_ (mm)	*S*_*sv*_ (mm)
Li et al.^6^	0–18.37	15.2	80	52.4	52.6	6(/)	80(/)
Wu^35^	0–6.1	15.2	50	92.4	55.7	6	100

“/” in the bracket means the specimen without stirrups.

**Table 2:** The conversion relationship among *f*_*cu*_, *f*_*c*_ and *f*_*t*_^44^.

*f*_*cu*_ (MPa)	15	20	25	30	35	40	45	50	55	60	65	70	75	80
*f*_*c*_ (MPa)	7.2	9.6	11.9	14.3	16.7	19.1	21.1	23.1	25.3	27.5	29.7	31.8	33.8	35.9
*f*_*t*_ (MPa)	0.91	1.10	1.27	1.43	1.57	1.71	1.80	1.89	1.96	2.04	2.09	2.14	2.18	2.22

**Table 3:** Experimental and theoretical values of ultimate average bond strength under different corrosion rate of steel strands.

Literatures	Specimen number	Corrosion rate (%)	Ultimate average bond strength (MPa)		*τ*_*exp*_/*τ*_*cal*_
			Experimental value(*τ*_*exp*_)	Theoretical value (*τ*_*cal*_)	
Li et al.^6^	BZ0-C1	0	11.16	10.19	1.094
	BZ1-C2	0.86	10.52	11.15	0.943
	BZ2-C2	2.36	10.22	10.68	0.957
	BZ3-C2	6.14	7.77	8.62	0.900
	BZ4-C1	11.76	5.07	6.56	0.773
	BZ0-S	0	13.74	10.91	1.26
	BZ1-S	0.98	11.37	12.15	0.936
	BZ3-S	7.42	7.54	8.25	0.914
	BZ4-S	11.49	5.93	6.68	0.887
	BZ6-S	18.37	2.54	2.04	1.25
	BP0-C1	0	10.92	10.20	1.07
	BP3-C2	2.11	11.78	10.78	1.092
	BP4-C1	5.87	8.48	8.69	0.976
	BP5-C1	7.86	6.42	8.32	0.772
	BP0-S2	0	12.87	10.91	1.18
	BP1-S1	0.1	15.38	11.92	1.291
	BP2-S1	1.1	14.12	12.15	1.162
	BP2-S2	1.86	12.7	11.92	1.066
	BP3-S1	3.36	10.34	11.19	0.925
	BP4-S1	4.61	9.76	10.43	0.935
	BP5-S1	8.34	7.11	7.95	0.894
	BP6-S1	14.49	3.75	5.02	0.747
Wu^35^	LB0	0	8.59	8.30	1.035
	LB1	0	7.72	8.69	0.889
	LB2	1.2	9.31	9.36	0.994
Wu^35^	LB3	1.6	10.23	12.17	0.841
	LB4	2.0	12.17	12.51	0.973
	LB5	2.7	13.06	9.80	1.332
	LB6	4.2	10.17	10.40	0.978
	LB7	6.1	6.25	8.88	0.704

**Figure 8:**
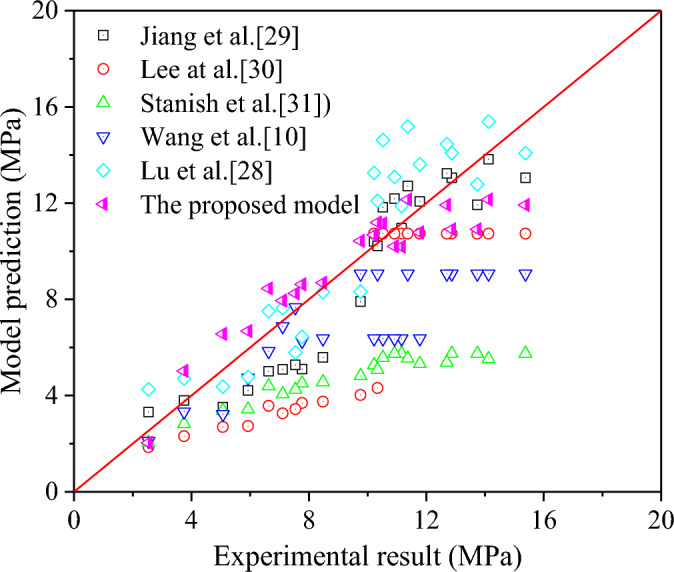
Comparison between experimental results and prediction of existing empirical models and new model.

In addition, the experimental results from the reference^6^ were compared with the prediction by the proposed model and the existing models, including the models proposed by Jiang et al.^45^, Lee et al.^46^ and Stanish et al.^47^ for the corroded steel bar, as well as, the model proposed by Wang et al.^20^ and Lu et al.^43^ for the corroded steel strands. The comparison between the experimental results and the predictions by these models is illustrated in Fig. 8. Also, Table 4 gives the average absolute error (AAE), mean square error (MSE), and standard deviation (SD) of these models to predict the bond strength between the corroded strand and concrete. It can be seen from the Fig. 8 and Table 4 that the proposed model exhibits good accuracy and consistency in predicting the test results of the bond strength between the corroded strand and concrete. Besides, the AAE, MSE and SD for the overall ratios of predicted results by the proposed model to experimental results are 0.133, 1.959 and 0.164 respectively. Except that SD of the proposed model is slightly larger than the existing models proposed by Stanish et al.^47^ and Wang et al.^20^, other statistical indicators are significantly better than other models. This indicates that the proposed analytical model can well predict the bond strength between corroded steel strand and concrete.

**Table 4:** Existing empirical models and new model to predict the bond strength after corrosion.

Literatures	AAE	MSE	SD
Jiang et al.^29^	0.155	2.322	0.183
Lee et al.^30^	0.310	10.004	0.226
Stanish et al.^31^	0.464	28.596	0.116
Wang et al.^10^	0.256	10.911	0.144
Lu et al.^28^	0.188	3.347	0.221
The proposed model	0.133	1.959	0.164

## Conclusion

An analytical model is proposed to investigate the bond strength between the corroded strand and concrete for the pull-out failure case. The following conclusions can be drawn:

- 1. The proposed bond strength model between corroded strand and concrete is derived based on the theory of elastic thick-walled cylinder. Factors that affect the bond strength, such as corrosion expansion of strand rust, the gripping effect of the concrete and friction coefficient have been considered in the model.
- 2. A comparison of results between the perdition and experimental data from the literature shows that the proposed model exhibits well performance in predicting the ultimate bond strength between corroded strand and concrete. The mean and standard deviation of the ratio between prediction and test results are 0.992 and 0.162, respectively.
- 3. The prediction result of the bond strength model is affected by the degree of strand corrosion. When the corrosion rate of steel strands is < 6.24%, the average ratio of the experimental result to the calculated value is 1.023, the analytical model underestimates the ultimate bond strength in the pull-out specimen. On the contrary, the analytical model will overestimate the ultimate bond strength of the specimen. The proposed analytical model is not sensitive to the influence of the drawing method.

## Data Availability

The data are presented in the manuscript. Furthermore, the detailed calculation process data are available from the corresponding author upon reasonable request.
